# Effects of Ketamine vs. Midazolam in Adolescent Treatment Resistant Depression

**DOI:** 10.3390/ph17121627

**Published:** 2024-12-04

**Authors:** Andrea Macejova, Veronika Kovacova, Ingrid Tonhajzerova, Zuzana Visnovcova, Nikola Ferencova, Zuzana Mlyncekova, Tomas Kukucka, Igor Ondrejka

**Affiliations:** 1 Clinic of Psychiatry, Jessenius Faculty of Medicine in Martin, Comenius University in Bratislava, University Hospital Martin, Kollarova 2, 03601 Martin, Slovakia; macejova5@uniba.sk (A.M.); kovacova400@uniba.sk (V.K.); ingrid.tonhajzerova@uniba.sk (I.T.); mlyncekova3@uniba.sk (Z.M.); kukucka17@uniba.sk (T.K.); 2 Department of Physiology, Jessenius Faculty of Medicine in Martin, Comenius University in Bratislava, Mala Hora 4C, 03601 Martin, Slovakia; nikola.ferencova@uniba.sk; 3 Biomedical Centre Martin, Jessenius Faculty of Medicine in Martin, Comenius University in Bratislava, Mala Hora 4D, 03601 Martin, Slovakia; zuzana.visnovcova@uniba.sk

**Keywords:** adolescent age, novel antidepressants, ketamine, midazolam, treatment-resistant depression

## Abstract

**Background:** Adolescent treatment resistant depression (TRD) is increasing in recent years. While ketamine showed rapid antidepressant effects in adult TRD studies, research on its effectiveness in adolescents is limited. **Methods:** This study examines the effects of intravenous ketamine vs. midazolam on depressive and anxiety symptomatology assessed by the Montgomery–Åsberg Depression Rating Scale (MADRS), Hamilton Anxiety Rating Scale (HAM-A), and Children’s Depression Inventory (CDI) at two time points—2 h after initial infusion (T0+2h) and 24 h after the end of the treatment (Te+24h) in a sample of 55 adolescent TRD females (27 receiving ketamine, 28 midazolam). **Results:** At T0+2h, within-group comparisons revealed a significant reduction in MADRS and HAM-A scores compared to baseline in the ketamine and midazolam groups. At Te+24h, both groups demonstrated similar significant reductions in MADRS, HAM-A, and CDI scores compared to baseline. The MADRS assessment in the ketamine group showed 33% and 59% responders, and in the midazolam group, 14% and 46% responders at T0+2h and Te+24h, respectively. HAM-A evaluation in the ketamine group revealed 33% and 56% responders, and in the midazolam group, 11% and 39% responders at T0+2h and at Te+24h, respectively. CDI rating discovered 11% and 44% responders in the ketamine group and 4% and 21% responders in the midazolam group at T0+2h and Te+24h, respectively. Moreover, inner tension significantly decreased in ketamine compared to the midazolam group at Te+24h. **Conclusions:** Ketamine showed a reduction in depressive and anxiety symptoms during a short-term period with particular efficacy in alleviating inner tension over midazolam, suggesting its potential advantages in specific symptom relief in rarely studied adolescent TRD.

## 1. Introduction

Major depressive disorder (MDD) represents an important public health concern with significantly increasing incidence in recent years, especially among adolescents [1]. More specifically, about 34% of individuals aged 10 to 19 report depressive symptoms, with a lifetime MDD prevalence estimated at 19% [1,2]. MDD in adolescence is nearly twice as common in females as in males [3] and is closely associated with an increased risk of suicide [4] and significant disability, characterized by impaired social and academic functioning [5,6], heightened likelihood of developing comorbid anxiety disorders, a greater risk of later MDD, early pregnancy in females, and substance abuse [7]. As a result, adolescent MDD is considered a significant global health problem, notably contributing to increased morbidity, mortality, and economic burden [6,8]. Importantly, approximately 40% of depressive adolescents show a lack of response to standard evidence-based treatment [9], such as selective serotonin reuptake inhibitors (SSRIs) and/or psychotherapeutic approaches [6,10]. The failure to achieve a reduction in depressive symptoms by more than 50% within a two-month period of pharmacological treatment at a sufficient therapeutic dose (i.e., 40 mg fluoxetine daily or its equivalent) or psychotherapy (8 to 16 sessions) in children and adolescents is defined as treatment resistant depression (TRD) [6,11]. However, the management of TRD is highly challenging, especially in adolescents with a few therapeutic options, including repetitive transcranial magnetic stimulation (rTMS), electroconvulsive therapy (ECT) [12], and nowadays increasingly discussed ketamine.

Ketamine is a non-competitive antagonist of the *N*-methyl-d-aspartate (NMDA) receptors, initially developed and utilized as an anesthetic agent in the fields of anesthesiology and pain management [13]. The revolutionary discovery of ketamine’s antidepressant potential in humans, when administered in subanesthetic doses (0.5 mg/kg), was made in 2000 [14]. Ketamine has been characterized by rapid-onset antidepressant and anti-suicidal effects manifesting within a few hours after the administration of a single ketamine infusion and lasting for approximately one to two weeks [15,16,17,18]. Moreover, current data from adult TRD studies consistently suggest that up to six low-dose ketamine infusions, administered once, twice, or three times per week, can produce a more robust antidepressant effect and effectively sustain and extend the treatment response [16,19,20]. However, the studies regarding ketamine’s efficacy in adolescent TRD are still limited.

In this context, the studies regarding the evaluation of the ketamine’s effectiveness should be placebo-controlled due to high placebo response rates in pediatric depression [21]. In this study, midazolam, as a short-acting benzodiazepine agent, was selected as an active comparator to ketamine according to its psychoactive properties of non-specific behavioral effects such as sedation or disorientation similar to ketamine and its pharmacokinetic characteristics similar to those of ketamine, such as fast onset of action and short elimination half-life [15]. The selection of midazolam was inspired by Murrough et al. [22], who were the first to use midazolam as an active comparator in ketamine trials in adult TRD. Further, the design of a two-week period of ketamine and midazolam administration during the initial phase of the patients’ hospitalization in a psychiatric ward was based on a protocol used in previous studies [16,23]. In this study, only females were included due to the higher prevalence of depression in adolescent girls compared to boys to minimize gender-based variability in the treatment response. Therefore, we aimed to investigate the antidepressant and anxiolytic efficacy of intravenous ketamine vs. midazolam in rarely studied adolescent females with TRD.

## 2. Results

Subject’s descriptive and clinical characteristics are summarized in the Table 1.

### 2.1. Primary Outcomes: Between-Group Comparisons

The nonparametric repeated-measurement ANOVA revealed no significant effect of group (ketamine and midazolam) in MADRS (χ^2^[1] = 0.23, *p* = 0.632, η^2^*p* = 0.004), HAM-A (χ^2^[1] = 0.02, *p* = 0.902, η^2^*p* = 0.001), and CDI scores (χ^2^[1] = 0.06, *p* = 0.815, η^2^*p* = 0.001). Moreover, no significant effect of group was found in responders’ proportions in MADRS (χ^2^[1] = 0.23, *p* = 0.632, η^2^*p* = 0.047), HAM-A (χ^2^[1] = 0.02, *p* = 0.902, η^2^*p* = 0.058), and CDI scores (χ^2^[1] = 0.06, *p* = 0.815, η^2^*p* = 0.061).

### 2.2. Secondary Outcomes: Within-Group Comparisons

The nonparametric repeated measurement ANOVA revealed the significant effect of time (T0, T0+2h, Te+24h) for MADRS (χ^2^[2] = 109.9, *p* < 0.001, η^2^*p* = 0.675), HAM-A (χ^2^[2] = 61.3, *p* < 0.001, η^2^*p* = 0.536), and CDI scores (χ^2^[2] = 29.4, *p* < 0.001, η^2^*p* = 0.357). Moreover, the mixed effect of group × time was found for MADRS score (χ^2^[2] = 5.0, *p* = 0.001, η^2^*p* = 0.086).

**Figure 1:**
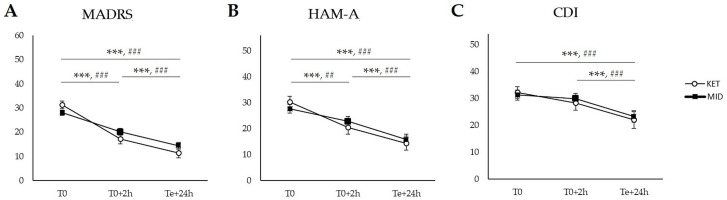
Effect of time (T0, T0+2h, Te+24h) in ketamine and midazolam groups. (**A**) MADRS score; (**B**) HAM-A score; and (**C**) CDI score. Abbreviations: T0—baseline, i.e., time point before treatment; T0+2h—time point representing two hours after initial infusion; Te+24h—time point representing twenty-four hours after last infusion; MADRS—the Montgomery–Åsberg Depression Rating Scale; CDI—the Children’s Depression Inventory; and HAM-A—the Hamilton Anxiety Rating Scale. Data are expressed as mean and SEM. White circle indicates ketamine group; black square indicates midazolam group; symbol * represents significant differences in ketamine group, symbol # represents significant differences in midazolam group (##: statistical significance at level *p* < 0.01, ***, ###: statistical significance at level *p* < 0.001).

In the ketamine group, the nonparametric Durbin–Conover post hoc test revealed significantly reduced MADRS and HAM-A scores in T0+2h compared to T0 (*p* < 0.001 for both). Next, MADRS, CDI, and HAM-A scores were significantly reduced in Te+24h compared to T0 (*p* < 0.001 for all). Lastly, MADRS, CDI, and HAM-A scores were significantly reduced in Te+24h compared to T0+2h (*p* < 0.001 for all). All results are summarized in Figure 1.

In the midazolam group, the post hoc analysis revealed significantly reduced MADRS and HAM-A scores in T0+2h compared to T0 (*p* < 0.001, *p* = 0.001, respectively). Next, MADRS, CDI, and HAM-A scores were significantly reduced in Te+24h compared to T0 (*p* < 0.001 for all). Lastly, MADRS, CDI, and HAM-A scores were significantly reduced in Te+24h compared to T0+2h (*p* < 0.001 for all). All results are summarized in Figure 1.

**Figure 2:**
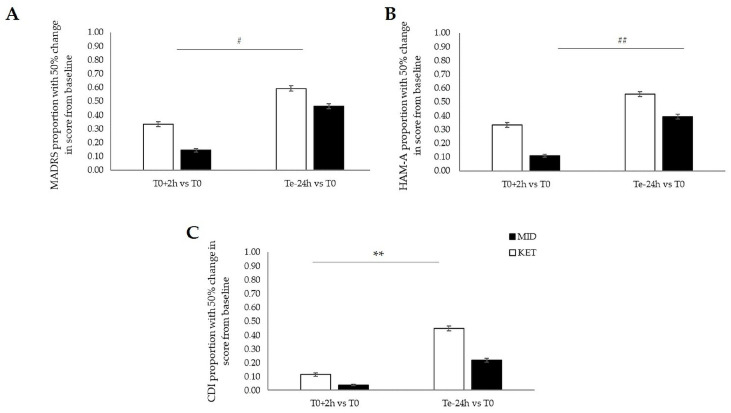
Responders’ proportion (≥50% improvement) in ketamine and midazolam groups. (**A**) MADRS score; (**B**) HAM-A score; and (**C**) CDI score. Abbreviations: T0—baseline, i.e., time point before treatment; T0+2h—time point representing two hours after initial infusion; Te+24h—time point representing twenty-four hours after last infusion; MADRS—the Montgomery–Åsberg Depression Rating Scale; CDI—the Children’s Depression Inventory; and HAM-A—the Hamilton Anxiety Rating Scale. Data are expressed as mean and SEM. The white column indicates the ketamine group; the black column indicates the midazolam group; symbol * represents significant differences in the ketamine group, symbol # represents significant differences in the midazolam group (#: statistical significance at level *p* < 0.05, **, ##: statistical significance at level *p* < 0.01).

Moreover, from the aspect of responders’ proportion evaluated by MADRS score with the cutoff point set at 50% change (decline) in score from baseline, 9 (33%) of 27 TRD adolescents at T0+2h and 16 (59%) of 27 TRD adolescents at Te+24h met response criteria in the ketamine group. Regarding the midazolam group, 4 (14%) of 28 TRD adolescents at T0+2h and 13 (46%) of 28 TRD adolescents met response criteria. Nonparametric repeated-measured ANOVA test revealed a significant effect of time (T0, T0+2h, and Te+24h) in the number of responders’ in the MADRS score (χ^2^[1] = 14.21, *p* < 0.001, η^2^*p* = 0.211). Post hoc Durbin–Conover test found a significantly increased number of responders at Te+24h compared to T0+2h in the midazolam group (*p* = 0.026). Next, responders’ proportion was measured by HAM-A score with the cutoff point set at 50% change (decline) in score from baseline; 9 (33%) of 27 TRD adolescents at T0+2h and 15 (56%) of 27 TRD adolescents met response criteria in the ketamine group, and 3 (11%) of 28 TRD adolescents at T0+2h and 11 (39%) of 28 TRD adolescents at Te+24h met response criteria in the midazolam group. ANOVA test revealed a significant effect of time (T0, T0+2h, and Te+24h) in the number of responders’ in the HAM-A score (χ^2^[1] = 18.11, *p* < 0.001, η^2^*p* = 0.255), and the post hoc Durbin–Conover test found a significantly increased number of responders at Te+24h compared to T0+2h in the midazolam group (*p* = 0.007). In addition, responders’ proportion evaluated by CDI score with the cutoff point set at 50% change (decline) in score from baseline: 3 (11%) of 27 TRD adolescents at T0+2h and 12 (44%) of 27 TRD adolescents met response criteria in the ketamine group, and 1 (4%) of 28 TRD adolescents at T0+2h and 6 (21%) of 28 TRD adolescents at Te+24h met response criteria in the midazolam group. ANOVA test revealed a significant effect of time (T0, T0+2h, and Te+24h) in the number of responders’ in the CDI score (χ^2^[1] = 18.89, *p* < 0.001, η^2^*p* = 0.263), and the post hoc Durbin–Conover test found a significantly increased number of responders at Te+24h compared to T0+2h in the ketamine group (*p* = 0.001). All results of responders’ proportions are summarized in Figure 2.

Finally, regarding individual MADRS symptoms, 1 (reflecting inner tension) of 10 symptoms showed a significant effect of group, all 10 symptoms showed a significant effect of time, and 3 (indicating apparent sadness, inner tension, and suicidal ideations) of 10 symptoms showed a significant mixed effect of time × group. Next, in the CDI individual symptoms assessment, all 5 symptoms showed a significant effect of time. Lastly, on the individual HAM-A symptoms, 1 (related to fears) of 14 symptoms showed a significant effect of group, all 14 symptoms showed a significant effect of time, and 1 (related to fears) of 14 symptoms showed a significant mixed effect of time × group. Detailed results evaluating individual symptoms from MADRS, HAM-A, and CDI scales are summarized in Supplementary Data.

## 3. Discussion

Surprisingly, this study revealed a comparable antidepressant and anxiolytic effect evaluated by the MADRS, HAM-A, and CDI total scores of both ketamine and midazolam. Several mechanisms are suggested.

The antidepressant and anxiolytic effects of ketamine can be driven through its action on multiple neurotransmiter systems and neural circuits. Ketamine primarily antagonizes NMDA receptors, resulting in the normalization of the glutamate signaling, activation of α-amino-3-hydroxy-5-methyl-4-isoxazolepropionic acid (AMPA) receptors stimulating the release of the brain-derived neurotrophic factor (BDNF), and activation of the mechanistic target of rapamycin (mTOR) signaling. This glutamatergic cascade triggers rapid synaptic remodeling and neuroplasticity. BDNF represents a protein promoting synaptic growth and neural resilience, while mTOR, as a signalling molecule involved in protein synthesis and cellular growth, contributes to the rebuilding of weakened synaptic connections. The increase in synaptic plasticity results in the formation of new neural circuits, particularly in the prefrontal cortex as a key brain region for improved emotional regulation, cognitive flexibility, and more adaptive responses to stress and anxiety-provoking situations. In summary, these mechanisms are believed to help improve stress adaptability and emotional regulation [17,24]. Noteworthy, the rapid antidepressant and anxiolytic effect of ketamine involves NMDA receptor antagonism and AMPA receptor activation, while sustained effects of ketamine contributing to improvements in mood and emotional resilience can be driven through its long-term effects on BDNF and mTOR signalling.

Unexpectedly, the study revealed a remarkable antidepressant effect of midazolam. The primary mechanism of midazolam’s effects can be explained by its action on GABA receptors in the brain. Specifically, midazolam binds to specific sites on the GABA-A receptors, which represent ligand-gated chloride ion channels, resulting in an allosteric modification that increases the affinity of the receptor for GABA as the main inhibitory neurotransmitter in the CNS. The more effective GABA binding to these receptors enhances the chloride ion channels opening and an influx of chloride ions into neurons, causing the hyperpolarization of the neurons, resulting in the reduction in neuronal excitability, which can manifest in sedative, anxiolytic, muscle relaxant, and anticonvulsant effects [25]. From this perspective, the antidepressant effect of midazolam observed in our study may be most likely attributed to indirect influences such as alleviating the overlapping anxiety symptoms or potential placebo effects. More specifically, as an active placebo, midazolam exhibits noticeable psychoactive effects influencing the patients’s overall perception of mood improvement [26,27]. In this line, the placebo response tends to be more pronounced, particularly in pediatric major depression characterized by higher suggestibility, differential neurotransmitter system maturation, and gender or developmental differences [28]. Additionally, the short follow-up period, during which patients were in a “protected hospital environment” associated with consistent psychological support, may have contributed to positive effects of midazolam on depressive symptomatology found in TRD adolescent females. On the contrary, a recent systematic review and meta-analysis of randomized controlled trials showed that benzodiazepines used as monotherapy could be more effective than placebo in the short-term treatment of depression [29]. Therefore, further research in this area is warranted.

From the aspect of clinician-rated the MADRS, we found a 33% response rate after initial infusion and a 59% response rate after the end of the treatment (sixth infusion) in the ketamine group and a 14% response rate after initial infusion associated with a 46% response rate after the end of the treatment in the midazolam group. Moreover, HAM-A evaluation revealed a 33% response rate after initial infusion and a 56% response rate after the end of the treatment in the ketamine group, and an 11% response rate after initial midazolam infusion and a 39% response rate after the end of the midazolam administration. The self-rated CDI pointed to an 11% response rate after initial infusion and a 44% response rate after the end of the treatment in the ketamine group, while a 4% response rate after initial infusion and a 21% response rate after the end of the treatment were in the midazolam group. These findings can potentially indicate better clinical response to ketamine in adolescent TRD patients, although no significant between-group differences were found. Previous study regarding MDD adolescent patients reported 54% responders assessed by MADRS criteria to esketamine treatment on the second post-treatment day, associated with an increasing 87% responders on the 25th post-treatment day [30]. In this context, future studies regarding MDD severity and duration are needed to better reflect the clinical response in MDD adolescents.

Lastly, the individual scale’s items analysis revealed that 9 of 10 MADRS symptoms, 2 of 5 CDI symptoms, and 11 of 14 HAM-A symptoms significantly improved after the initial infusion of ketamine, while 8 of 10 MADRS symptoms, 0 of 5 CDI symptoms, and 5 of 14 HAM-A symptoms significantly improved after the initial infusion of midazolam. On the other hand, repeated ketamine infusions resulted in the significant improvement of all symptoms regarding MADRS, CDI, and HAM-A, while repeated midazolam infusions resulted in the significant improvement of all MADRS and CDI symptoms associated with 13 HAM-A symptoms (from a total of 14 symptoms). Importantly, the between-group analysis of the study pointed to a better effect of ketamine on inner tension (MADRS_3) compared to midazolam at the end of treatment following repeated infusions, indicating potential superior anxiolytic efficacy of ketamine over midazolam, which is in line with previous research indicating assumed better efficacy of ketamine on stress-related depression [31]. Since inner tension often arises from unresolved emotional conflict, chronic stress, and underlying depression or anxiety, ketamine can play a crucial role in its reduction by modulating key neurotransmitter systems, promoting neuroplasticity, and disrupting maladaptive thought patterns [32,33,34]. Moreover, the majority of anxiety symptoms measured through clinician-administered HAM-A significantly improved after the initial ketamine infusion, highlighting its rapid onset of anxiolytic effect consistent with the findings of previous studies [35,36,37,38,39]. The significant improvements suggest that ketamine’s mechanism reduced anxiety symptoms within a concise time frame with the following enhancement of TRD-linked symptoms after repeated infusions resulting in the reduction in all HAM-A parameters at the end of the treatment. Moreover, the likelihood of occurrence of suicidal events in depressive adolescents correlating with high non-response to antidepressant treatment becomes an important clinical concern [40,41]. In this context, this study evaluated the suicidal ideation via the MADRS_10 item, revealing comparable, significantly reduced suicidal ideation after the initial and repeated infusions in both the ketamine and midazolam groups. However, no significant difference between ketamine and midazolam was revealed. Since ketamine is well-known for its anti-suicidal effects [42,43,44], midazolam has not been aligned with direct anti-suicidal effects. Therefore, the absence of a significant difference between the ketamine and midazolam groups in reducing suicidal ideation scores in this study can be potentially explained by the indirect influence of midazolam’s immediate sedative and anxiolytic effects contributing to mood improvements and consequently alleviating suicidal thoughts [31,45]. In this context, the correlations between mood improvements and reductions in suicidal thinking were found with both ketamine and midazolam, a finding that is in accordance with our results [46]. In ontrast, ketamine has been shown to more rapidly reduce suicidal ideation within one day and for up to one week of administration compared to control conditions, including the use of midazolam [44]. Therefore, future research is required for more in-depth exploration of the complex relationships in the mechanisms of anti-suicidal effects of ketamine vs. midazolam.

### Limitations

This study is limited by a studied group with only a single-gender cohort (females). Next, the study is limited by the potential effect of the “protective“ hospital environment with minimalization of external stressors and receiving psychological support, which should be taken into account in the interpretation of our findings. Another limitation of this study is the short two-week treatment period; therefore, long-term observation is needed to assess the efficacy and safety of ketamine and midazolam in TRD adolescent depression. Therefore, further research on a larger sample of patients with respect to gender and detailed classification of the different MDD specifiers according to DSM-5, including longer follow-up treatment periods, is needed, particularly at the vulnerable adolescent age. Moreover, the comparison with other antidepressants or placebo treatments could provide more information about TRD-linked treatment in adolescent age. Addressing these limitations could improve the understanding of both treatments and support their effective application in clinical practice.

## 4. Materials and Methods

### 4.1. Ethics Approval

The authors claim that all procedures contributing to this study meet the ethical standards of the national and institutional committees and the Helsinki Declaration of 1975, as revised in 2008. The study protocol was approved by the Ethics Committee of the Jessenius Faculty of Medicine in Martin, Comenius University in Bratislava, the Slovak Republic (approval no. 56/2021, dated 27 September 2021), and by the Ethics Committee of University Hospital Martin, the Slovak Republic (approval no. 143/2021, dated 10 September 2021). All patients’ legal representatives were fully informed about the study and gave their written consent.

### 4.2. Study Design and Patients

Patients were enrolled in the study, which was part of the research under the Jessenius Faculty of Medicine at Comenius University in Martin, Slovakia, from September 2021 to June 2024. The patients suffering from a major depressive disorder (MDD) were recruited from the inpatients admitted to the Psychiatric Clinic of Jessenius Faculty of Medicine and University Hospital in Martin. The primary diagnosis of MDD, severe intensity, and without psychotic symptoms was classified and confirmed by a thorough clinical investigation based on the unstructured diagnostic interview by two independent certified child and adolescent psychiatrists and a staff child/adolescent psychiatrist, according to the Diagnostic and Statistical Manual of Mental Disorders criteria [47]. None of the patients were diagnosed with reactive depression, adjustment disorder with anxiety and depressed mood, or other similar comorbidities. Inclusion criteria were as follows: adolescent females aged 12 to 18 years; diagnosis of Major Depressive Disorder (MDD) according to DSM-5 criteria, a severe depressive episode, confirmed by independent clinical evaluations by two certified child and adolescent psychiatrists; and treatment resistance. The TRD was established based on previous evidence-based treatment protocols for adolescent depression [6,11]. Specifically, to be indicated as TRD, adolescents must have inadequate response to at least one prior trial of standard antidepressant medication (SSRI) at a sufficient therapeutic dose (equivalent to 40 mg of fluoxetine daily) for eight weeks. Exclusion criteria included a personal history of psychotic illness, manic episodes, comorbid anxiety, substance abuse or dependence, a positive pregnancy test, known allergic or adverse reactions to the study medications, and any somatic conditions that contraindicate the use of both medications, such as untreated hypertension, glaucoma, increased intracranial pressure, a history of seizures, severe respiratory failure, acute respiratory distress, or myasthenia gravis. Of the 70 patients who were initially enrolled, 15 dropped out due to not meeting inclusion criteria or filling exclusion criteria (*n* = 7), adverse effects (*n* = 3), and missing sufficient data (*n* = 5). The final sample comprised 55 female adolescent patients suffering from TRD aged 13 to 17 years, with 27 TRD patients involved in the ketamine group (aged: 14.9 ± 1.5 SD years) and 28 in the midazolam group (aged: 15.2 ± 1.3 SD years). The post-enrollment patient allocation was randomized in a 1:1 ratio, and participants were blinded to group assignments.

**Figure 3:**
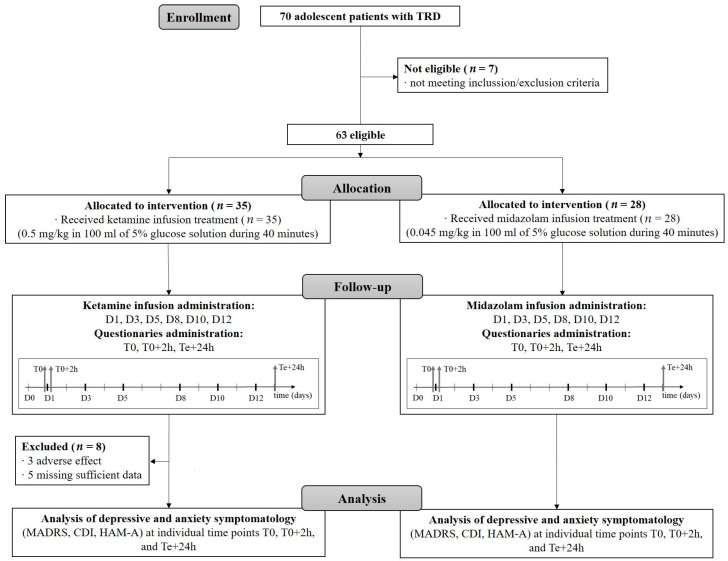
Study flow chart. TRD–treatment resistant depression, D1, D3, D5, D8, D10, and D12—day one, three, five, eight, ten, and twelve, respectively, after treatment start; T0—baseline, i.e., time point before treatment; T0+2h—time point representing two hours after initial infusion; Te+24h—time point representing twenty-four hours after last infusion, MADRS—the Montgomery–Åsberg Depression Rating Scale, HAM—A–the Hamilton Anxiety Rating Scale, CDI—the self-report Children’s Depression Inventory.

Each patient received a total amount of six infusions of either ketamine or midazolam over a two-week treatment period. Infusions were administered three times per week on alternating days (Day 1, Day 3, and Day 5 during the first week; Day 8, Day 10, and Day 12 during the second week) (see Figure 3).

### 4.3. Study Procedures

Firstly, each participant in the study undergoes a somatic examination, which includes the collection of peripheral venous blood samples for basic biochemical examinations, an electrocardiogram (ECG) examination, urine toxicology screening, and a pregnancy test. Inpatients fasted for 8 h and abstained from drinking for 2 h prior to the administration of the infusion. Since the entry criterion for the study was the lack of efficacy of prior antidepressant treatment, the participants were discontinued from their prescribed antidepressant therapy during the ketamine/midazolam treatment period, with infusions starting on the second or third day of hospitalization.

On the morning of the treatment day, a peripheral venous catheter on the non-dominant hand was secured. Ten minutes before the treatment, vital signs, including blood pressure and heart rate, were monitored using an automated oscillometric device (OMRON M6 Comfort, Kyoto, Japan), and blood oxygen saturation was measured using digital pulse oximetry. Consequently, ketamine (racemic mixture, Calypsol 50 mg/mL solution for injection) at a dose of 0.5 mg/kg of actual body weight or midazolam (Midazolam Accord 5 mg/mL infusion solution) at a dose of 0.045 mg/kg of actual body weight were administered. Both medications were diluted in 100 mL of 5% glucose solution and delivered using an infusion pump (B Braun Perfusor Compact Syringe Pump, Melsungen, Germany) over a 40 min period. The treatment doses were based on previous randomized controlled studies [21,22,48,49]. During the treatment, a supportive interview with the patient was conducted, primarily focused on managing any potential discomfort, anxiety, or stress during the medication administration. Changes in vital signs were monitored, with blood pressure, heart rate, and blood oxygen saturation recorded every fifteen minutes during the treatment and then every hour for two hours after the therapy. During the treatment, we ensure a comfortable and safe environment for the patient by eliminating any disturbing influences, such as loud noises or strong lighting. In the event of an emergency, the anesthesiology team and necessary equipment (including resuscitation devices, medications for high blood pressure, benzodiazepine antidotes, etc.) were readily available. Before the patient was discharged from the treatment room, clinical stability was required, defined as stable vital signs and a minimum score of 9 points on the modified Aldrete scoring system [50].

### 4.4. Psychometric Measures

The following validated and reliable rating scales in the adolescent population were used to assess symptoms of depression and anxiety before, during, and after treatment: the Montgomery–Åsberg Depression Rating Scale (MADRS) [51], the Hamilton Anxiety Rating Scale (HAM-A) [52], and the Children’s Depression Inventory (CDI) [53].

MADRS is a clinician-administered validated 10-item tool that measures the severity of depressive symptoms. It demonstrates psychometric properties comparable to other standardized measures of depression severity [54], and it is a sensitive tool with high inter-rater reliability (0.95) [55]. Widely used by clinicians, its main focus is on affective, cognitive, social, and somatic aspects of depression [51,54]. The individual items of the scale are as follows: apparent sadness (No. 1), reported sadness (No. 2), inner tension (No. 3), reduced sleep (No. 4), reduced appetite (No. 5), concentration difficulties (No. 6), lassitude (No. 7), inability to feel (No. 8), pessimistic thoughts (No. 9), and suicidal ideations (No. 10). The items are scored on a seven-point scale from 0, meaning none, to 6, meaning severe. The rater must decide whether the rating lies on the defined scale steps (0, 2, 4, 6) or between them (1,3,5). The overall score can range from 0 to 60, with a total score of 31 points or higher indicating severe depression [56].

HAM-A [52] is a clinician-administered 14-item scale for rating the severity of anxiety in adults, adolescents, and children with proven reliability, validity, and sensitivity [57]. Each of the 14 items targets a group of symptoms, and each cluster is rated on a scale of zero to four, with four being the most severe. The individual symptom groups are: anxious mood (No. 1), tension (No. 2), fears (No. 3), insomnia (No. 4), intellectual (No. 5), depressed mood (No. 6), somatic (muscular) (No. 7), somatic (sensory) (No. 8), cardiovascular symptoms (No. 9), respiratory symptoms (No. 10), gastrointestinal symptoms (No. 11), genitourinary symptoms (No. 12), autonomic symptoms (No. 13), behavior at interview (No. 14). The items address both psychic and somatic anxiety (physical complaints related to anxiety). The individual items are scored on a five-pointed scale from 0, meaning none, to 4, meaning a very severe symptom. The total score can range from 0 to 56 points, with a score of ≤17 indicating mild anxiety, 18–24 mild to moderate anxiety, 25–30 moderate to severe anxiety, and >30 very severe anxiety [58].

CDI is a 27-item validated self-assessment tool designed to evaluate cognitive, affective, and behavioral indicators of depression in children and adolescents (aged 7 to 17) [59]. Each item contains three statements from which the patient selects one, with responses scored from 0 to 2 points. According to Kovacs [53], a five-factor model can be applied, where individual areas are significantly intercorrelated: negative mood (CDI_A), interpersonal problems (CDI_B), ineffectiveness (CDI_C), anhedonia (CDI_D), and negative self-esteem (CDI_E). The individual areas are rated on a scale as follows: CDI_A from 0 to 12, CDI_B and CDI_C from 0 to 8, CDI_D from 0 to 16, and CDI_E from 0 to 10. The total score ranges from 0 to 54 points, with a clinical cut-off score for depression of 13 points [53], although other studies suggest a cut-off score of 19 points [60,61].

The MADSR and HAM-A scales were rated by trained investigators who were blinded to the group assignments of participants, ensuring objective and unbiased assessments. The quantification of depressive and anxiety symptomatology was assessed at three time points–baseline (T0), two hours after the initial infusion (T0+2h), and at the end of the treatment after repeated infusions (i.e., twenty-four hours after the last, sixth infusion–Te+24h). This assessment was conducted independently within each treatment arm, along with a comparative analysis between the ketamine and midazolam groups.

### 4.5. Outcomes

The primary outcomes were focused on the change in depressive/anxiety symptomatology assessed by MADRS, CDI, and HAM-A at T0+2h after the initial ketamine infusion and at Te+24h after repeated ketamine infusions (six in total) lasting two weeks in comparison to midazolam (i.e., between-group comparison).

The secondary outcomes were focused on the change in depressive/anxiety symptomatology using MADRS, CDI, and HAM-A to the initial and followed infusion treatment (i.e., T0 vs. T0+2h vs. Te+24h) separately in the ketamine and midazolam groups (i.e., within-group comparison).

Moreover, clinical response was defined as a ≥ 50% decrease in the MADRS [30], HAM-A [62], and CDI [63] scores from baseline.

### 4.6. Statistical Analysis

The data were explored and analyzed in jamovi version 1.6.9 (Sydney, Australia). Gaussian/non-Gaussian distribution of data was evaluated by the Shapiro–Wilk normality statistical test with the null hypothesis that the data distribution is parametric. The basic characteristics data were normally distributed; thus the parametric independent sample t-test was used for between group comparison. The questionnaire’s data and responders’ proportions were non-parametric; thus, the non-parametric repeated measurement analysis of variance (ANOVA) (Friedman test) with the Durbin–Conover post hoc test was used for the effect of group (ketamine group vs. midazolam group), the effect of time (T0, T0+2h, and Te+24h), and the mixed effect of group × time assessment. Next, the effect size for non-parametric repeated measures ANOVA was evaluated by partial eta-squared (η^2^*p*, ranging from 0 to 1). Threshold values for η^2^*p* are interpreted as small (0.01), medium (0.06), and large (0.14) powers [64]. Next, the sample size was estimated according to published data by [21], where the mean difference for the MADRS score between ketamine and midazolam groups at 24 h after infusion was equal to minus 8.69, SD = 15.08, and effect size = 0.78. After using this result in the following formula for effect size calculation [65]: 2 × (Z_α_ + Z_1−β_)^2^ × σ^2^/Δ^2^, where α level was 0.05, Z_α_, and Z_1−β_ at 90% power were constant 1.65 and 1.2816, respectively, σ represents SD, and Δ–mean differences between groups. The minimal sample size was 52, i.e., 26 probands per group. Subject characteristics data (except Adequate AD Trials per patient was expressed) were expressed as mean ± SEM; the parameter Adequate AD Trials per patient as median (interquartile range, IQR). Questionnaire’s data in figures were expressed as mean ± SEM. Value *p* < 0.05 was considered statistically significant.

## 5. Conclusions

Treatment resistant depression represents a serious problem, especially in adolescents with limited evidence for its effective management. Our study revealed that both ketamine and midazolam have shown a significant reduction in depressive and anxiety symptomatology during a short-term time period, with a particular superior effect of ketamine in alleviating inner tension over midazolam, suggesting its greater potential in specific symptom relief in rarely studied adolescent TRD. Further prospective controlled studies are required to provide the evidence-based effectiveness of newly developing drugs such as ketamine in pediatric TRD management.

## Figures and Tables

**Table 1:** Descriptive characteristics of the studied groups.

Parameter	Ketamine Group (*n* = 27)	Midazolam Group (*n* = 28)	*p*-Value
Average age (years)	14.9 ± 0.3	15.2 ± 0.2	0.494
BMI (kg/m^2^)	23.0 ± 1.2	23.3 ± 0.9	0.826
Sex	Only female	Only female	-
SBP (mmHg)	116.0 ± 2.3	112.0 ± 1.7	0.100
DBP (mmHg)	72.9 ± 1.8	71.2 ± 1.9	0.528
HR (bpm)	88.4 ± 2.7	87.9 ± 3.1	0.894
Blood oxygen saturation (%)	98.1 ± 0.2	98.0 ± 0.2	0.715
Adequate AD Trials per patient	2 (1, 2)	2 (1, 3)	0.458
Current AD medication	none	none	-
Previous AD treatment	Fluoxetine (*n* = 21)	Fluoxetine (*n* = 20)	-
	Sertraline (*n* = 12)	Sertraline (*n* = 14)	
	Fluvoxamine (*n* = 5)	Escitalopram (*n* = 7)	
	Trazodone (*n* = 5)	Trazodone (*n* = 6)	
	Vortioxetine (*n* = 4)	Vortioxetine (*n* = 4)	
	Escitalopram (*n* = 3)	Fluvoxamine (*n* = 2)	
	Sertraline (*n* = 12)	Citalopram (*n* = 1)	
	Citalopram (*n* = 2)	Sertraline (*n* = 14)	

*n*—number of patients, BMI—body mass index, AD—antidepressant, SBP—systolic blood pressure (mmHg), DBP—diastolic blood pressure (mmHg), HR—heart rate (beats per minute, bpm). Data (except Adequate AD Trials per patient) were expressed as mean ± SEM; the parameter Adequate AD Trials per patient was expressed as median (interquartile range). The value of *p* < 0.05 is considered statistically significant.

## Data Availability

Complete data are available upon reasonable request from the corresponding author.

## Supplementary Materials

The following supporting information can be downloaded at: https://www.mdpi.com/article/10.3390/ph17121627/s1, Figure S1: Between group (ketamine vs. midazolam) comparisons; (A) subscale of HAM-A No. 3 (fear); (B) subscales of MADRS No. 3 (inner tension); Figure S2: Effect of time (T0, T0+2h, Te+24h) within ketamine group. (A) MADRS parameters; (B) CDI parameters; (C) HAM-A parameters; Figure S3: Effect of time (T0, T0+2h, Te+24h) within midazolam group. (A) MADRS parameters; (B) CDI parameters; (C) HAM-A parameters.
