# Supplementary material for: Effects of Ketamine vs. Midazolam in Adolescent Treatment Resistant Depression
Source: Pharmaceuticals (Basel). 2024 Dec 4;17(12):1627. doi: 10.3390/ph17121627 (PMC11728720; doi:10.3390/ph17121627)
Supplement: Supplementary file 1 [file pharmaceuticals-17-01627-s001.zip › pharmaceuticals-3312339-supplementary.pdf]

SUPPLEMENTARY DATA

Between-Group Comparisons of Individual MADRS, HAM-A, and CDI Symptoms

From aspect of clinician-administrated questionnaire measuring the severity of anxiety (HAM-A), the nonparametric repeated measurement ANOVA revealed significant effect of group (ketamine and midazolam) for HAM-A_3 at T0 ($\chi^2[1] = 8.526$, $p = 0.004$, $\eta^2p = 0.158$). The nonparametric Durbin-Conover post hoc test revealed significantly increased HAM-A_3 related to fears at baseline in the ketamine group compared to the midazolam group ($p = 0.044$).

From aspect of clinician-administrated questionnaire measuring the severity of depressive symptoms (MADRS), the nonparametric repeated measurement ANOVA revealed the significant effect of group (ketamine vs midazolam) for MADRS_3 at Te+24h ($\chi^2[1] = 5.500$, $p = 0.019$, $\eta^2p = 0.102$). The nonparametric Durbin-Conover post hoc test revealed significantly decreased MADRS_3 related to inner tension at the end of the treatment (i.e. 24 h after the sixth infusion) in the ketamine group compared to the midazolam group ($p = 0.049$).

The results are summarized in Figure S1.

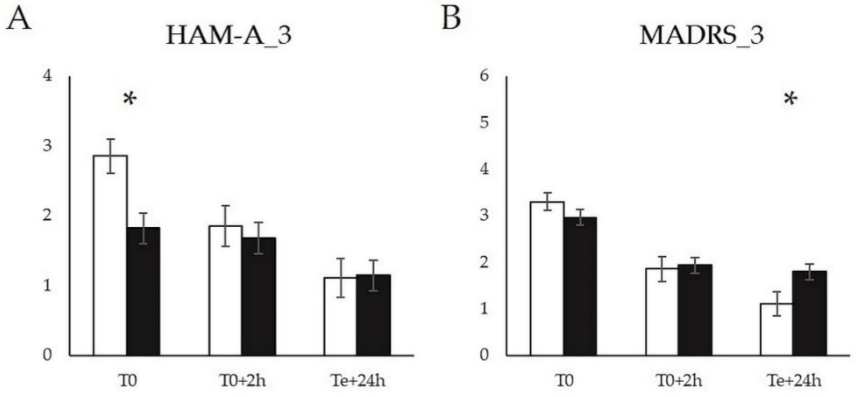

Figure S1. Between group (ketamine vs. midazolam) comparisons; (**A**) subscale of HAM-A No. 3 (fear); (**B**) subscales of MADRS No. 3 (inner tension). Abbreviations: T0—baseline, i.e. time point before treatment, T0+2h—time point representing two hours after initial infusion, Te+24h—time point representing twenty-four hours after last infusion, MADRS—the Montgomery-Åsberg Depression Rating Scale, and HAM-A—Hamilton Anxiety Rating Scale. White columns represent ketamine group, and black columns represent midazolam group. Data are expressed as mean and SEM. * represents statistical significance at level $p < 0.05$.

Within-Group Comparisons of Individual MADRS, HAM-A, and CDI Symptoms

From aspect of clinician-administrated questionnaire measuring the severity of depressive symptoms (MADRS), the nonparametric repeated measurement ANOVA revealed the significant effect of time (T0, T0+2h, Te+24h) for all evaluated MADRS items, i.e. MADRS_1 ($\chi^2[2] = 73.2$, $p < 0.001$, $\eta^2p = 0.580$), MADRS_2 ($\chi^2[2] = 62.8$, $p < 0.001$, $\eta^2p = 0.542$), MADRS_3 ($\chi^2[2] = 55.3$, $p < 0.001$, $\eta^2p = 0.511$), MADRS_4 ($\chi^2[2] = 19.4$, $p < 0.001$, $\eta^2p = 0.268$), MADRS_5 ($\chi^2[2] = 19.6$, $p < 0.001$, $\eta^2p = 0.270$), MADRS_6 ($\chi^2[2] = 36.3$, $p < 0.001$, $\eta^2p = 0.407$), MADRS_7 ($\chi^2[2] = 65.0$, $p < 0.001$, $\eta^2p = 0.551$), MADRS_8 ($\chi^2[2] = 20.4$, $p < 0.001$, $\eta^2p = 0.278$),

MADRS_9 ($\chi^2[2] = 44.2$, $p < 0.001$, $\eta^2p = 0.455$), and MADRS_10 ($\chi^2[2] = 77.2$, $p < 0.001$, $\eta^2p = 0.593$). Moreover, the mixed effect of group × time was found for MADRS_1 ($\chi^2[2] = 5.2$, $p = 0.007$, $\eta^2p = 0.090$), MADRS_3 ($\chi^2[2] = 4.7$, $p = 0.011$, $\eta^2p = 0.081$), and MADRS_10 ($\chi^2[2] = 3.2$, $p = 0.045$, $\eta^2p = 0.057$).

From aspect of self-assessment questionnaire measuring cognitive, affective, and behavioral indicators of depression (CDI), the nonparametric repeated measurement ANOVA revealed significant effect of time (T0, T0+2h, Te+24h) for following items: CDI_A ($\chi^2[2] = 21.8$, $p < 0.001$, $\eta^2p = 0.292$), CDI_B ($\chi^2[2] = 17.2$, $p < 0.001$, $\eta^2p = 0.246$), CDI_C ($\chi^2[2] = 17.2$, $p < 0.001$, $\eta^2p = 0.245$), CDI_D ($\chi^2[2] = 25.5$, $p < 0.001$, $\eta^2p = 0.325$), and CDI_E ($\chi^2[2] = 20.7$, $p < 0.001$, $\eta^2p = 0.281$).

From aspect of clinician-administrated questionnaire measuring the severity of anxiety (HAM-A), the nonparametric repeated measurement ANOVA revealed significant effect of time (T0, T0+2h, Te+24h) for HAM-A_1 ($\chi^2[2] = 27.6$, $p < 0.001$, $\eta^2p = 0.342$), HAM-A_2 ($\chi^2[2] =26.7$, $p < 0.001$, $\eta^2p = 0.335$), HAM-A_3 ($\chi^2[2] = 23.3$, $p < 0.001$, $\eta^2p = 0.305$), HAM-A_4 ($\chi^2[2] = 5.6$, $p = 0.005$, $\eta^2p = 0.095$), HAM-A_5 ($\chi^2[2] = 18.8$, $p < 0.001$, $\eta^2p = 0.261$), HAM-A_6 ($\chi^2[2] = 23.7$, $p < 0.001$, $\eta^2p = 0.309$), HAM-A_7 ($\chi^2[2] = 18.9$, $p < 0.001$, $\eta^2p = 0.263$), HAM-A_8 ($\chi^2[2] = 30.5$, $p < 0.001$, $\eta^2p = 0.365$), HAM-A_9 ($\chi^2[2] = 32.7$, $p < 0.001$, $\eta^2p = 0.381$), HAM-A_10 ($\chi^2[2] = 16.5$, $p < 0.001$, $\eta^2p = 0.238$), HAM-A_11 ($\chi^2[2] = 12.0$, $p < 0.001$, $\eta^2p = 0.185$), HAM-A_12 ($\chi^2[2] = 9.2$, $p < 0.001$, $\eta^2p = 0.148$), HAM-A_13 ($\chi^2[2] = 14.0$, $p < 0.001$, $\eta^2p = 0.208$), and HAM-A_14 ($\chi^2[2] = 9.9$, $p < 0.001$, $\eta^2p = 0.158$). Moreover, the mixed effect of group x time was found for HAM-A_3 ($\chi^2[2] = 5.1$, $p = 0.008$, $\eta^2p = 0.087$).

In ketamine group, the nonparametric Durbin-Conover post hoc test revealed significantly reduced MADRS_1, MADRS_2, MADRS_3, MADRS_4, MADRS_6, MADRS_7, MADRS_8, MADRS_9, MADRS_10, CDI_A, CDI_E, HAM-A_1, HAM-A_2, HAM-A_3, HAM-A_4, HAM-A_5, HAM-A_6, HAM-A_7, HAM-A_8, HAM-A_9, HAM-A_10, and HAM-A_13 in T0+2h compared to T0 ($p < 0.001$, $p < 0.001$, $p < 0.001$, $p = 0.033$, $p < 0.001$, $p < 0.001$, $p < 0.001$, $p < 0.001$, $p < 0.001$, $p = 0.041$, $p = 0.008$, $p = 0.004$, $p = 0.010$, $p = 0.029$, $p = 0.044$, $p = 0.006$, $p = 0.001$, $p = 0.003$, $p = 0.004$, $p = 0.001$, $p = 0.021$, $p = 0.001$, respectively). Next, MADRS_1, MADRS_2, MADRS_3, MADRS_4, MADRS_5, MADRS_6, MADRS_7, MADRS_8, MADRS_9, MADRS_10, CDI_A, CDI_B, CDI_C, CDI_D, CDI_E, HAM-A_1, HAM-A_2, HAM-A_3, HAM-A_4, HAMA-A_5, HAM-A_6, HAM-A_7, HAM-A_8, HAM-A_9, HAM-A_10, HAM-A_11, HAM-A_12, HAM-A_13, and HAM-A_14 were significantly reduced in Te+24h compared to T0 ($p < 0.001$, $p < 0.001$, $p < 0.001$, $p < 0.001$, $p < 0.001$, $p < 0.001$, $p < 0.001$, $p < 0.001$, $p < 0.001$, $p < 0.001$, $p < 0.001$, $p < 0.001$, $p < 0.001$, $p < 0.001$, $p < 0.001$, $p < 0.001$, $p < 0.001$, $p < 0.001$, $p = 0.009$, $p = 0.002$, $p < 0.001$, $p < 0.001$, $p < 0.001$, $p < 0.001$, $p < 0.001$, $p = 0.005$, $p < 0.001$, $p < 0.001$, $p = 0.005$, respectively). Lastly, MADRS_2, MADRS_3, MADRS_4, MADRS_5, MADRS_6, MADRS_7, MADRS_9, MADRS_10, CDI_B, CDI_C, CDI_D, HAM-A_2, HAM-A_3, HAM-A_8, and HAM-A_10 were significantly reduced in Te+24h compared to T0+2h ($p = 0.047$, $p = 0.010$, $p = 0.016$, $p = 0.002$, $p = 0.015$, $p < 0.001$, $p = 0.045$, $p = 0.020$, $p = 0.028$, $p < 0.001$, $p < 0.001$, $p = 0.020$, $p = 0.003$, $p = 0.040$, $p = 0.009$, respectively). All results are summarized in Figure S2.

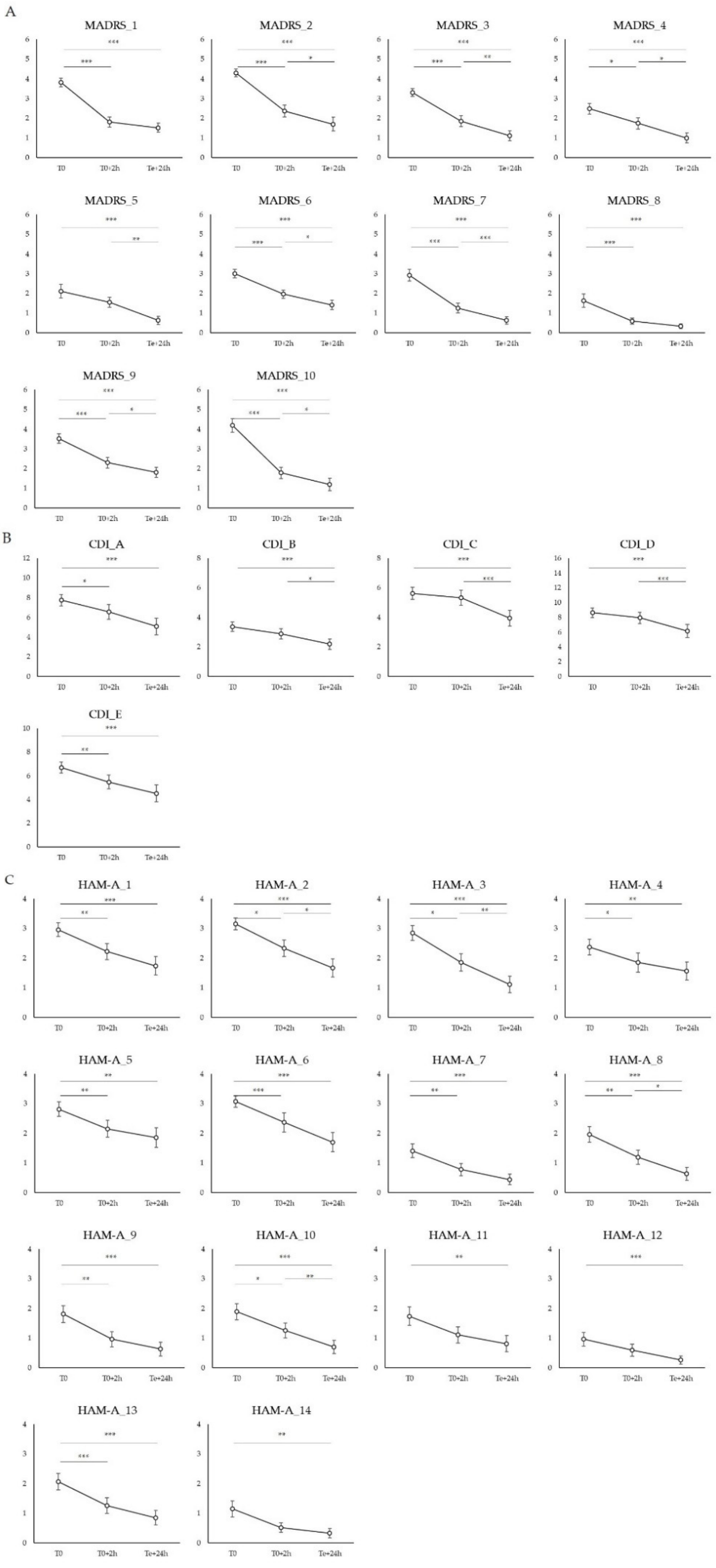
A
MADRS_1
MADRS_2
MADRS_3
MADRS_4
MADRS_5
MADRS_6
MADRS_7
MADRS_8
MADRS_9
MADRS_10
B
CDI_A
CDI_B
CDI_C
CDI_D
CDI_E
C
HAM-A_1
HAM-A_2
HAM-A_3
HAM-A_4
HAM-A_5
HAM-A_6
HAM-A_7
HAM-A_8
HAM-A_9
HAM-A_10
HAM-A_11
HAM-A_12
HAM-A_13
HAM-A_14
T0
T0+2h
Te+24h

Figure S2. Effect of time (T0, T0+2h, Te+24h) within ketamine group. (**A**) MADRS parameters; (**B**) CDI parameters; (**C**) HAM-A parameters. Abbreviations: T0—baseline, i.e. time point before treatment, T0+2h—time point representing two hours after initial infusion, Te+24h—time point representing twenty-four hours after last infusion, MADRS—the Montgomery-Åsberg Depression Rating Scale, CDI—the self-report Children's Depression Inventory, and HAM-A—Hamilton Anxiety Rating Scale. Data are expressed as mean and SEM. * represents statistical significance at level $p < 0.05$; ** represent statistical significance at level $p < 0.01$, *** represent statistical significance at level $p < 0.001$.

In midazolam group, the post hoc analysis revealed significantly reduced MADRS_1, MADRS_2, MADRS_3, MADRS_6, MADRS_7, MADRS_8, MADRS_9, MADRS_10, HAM-A_1, HAM-A_2, HAM-A_8, HAM-A_9 in T0+2h compared to T0 ($p < 0.001$, $p < 0.001$, $p < 0.001$, $p = 0.003$, $p < 0.001$, $p = 0.003$, $p = 0.013$, $p < 0.001$, $p < 0.001$, $p = 0.029$, $p < 0.001$, $p < 0.001$, respectively). Next, MADRS_1, MADRS_2, MADRS_3, MADRS_4, MADRS_5, MADRS_6, MADRS_7, MADRS_8, MADRS_9, MADRS_10, CDI_A, CDI_B, CDI_C, CDI_D, CDI_E, HAM-A_1, HAM-A_2, HAM-A_3, HAMA-A_5, HAM-A_6, HAM-A_7, HAM-A_8, HAM-A_9, HAM-A_10, HAM-A_11, HAM-A_12, HAM-A_13, and HAM-A 14 were significantly reduced in Te+24h compared to T0 ($p < 0.001$, $p < 0.001$, $p < 0.001$, $p < 0.001$, $p < 0.001$, $p < 0.001$, $p < 0.001$, $p < 0.001$, $p < 0.001$, $p < 0.001$, $p < 0.001$, $p < 0.001$, $p = 0.010$, $p < 0.001$, $p < 0.001$, $p < 0.001$, $p < 0.001$, $p = 0.005$, $p < 0.001$, $p < 0.001$, $p < 0.001$, $p < 0.001$, $p < 0.001$, $p = 0.001$, $p < 0.001$, $p = 0.046$, $p = 0.046$, $p = 0.002$, respectively). Lastly, MADRS_1, MADRS_4, MADRS_5, MADRS_6, MADRS_7, MADRS_9, MADRS_10, CDI_A, CDI_B, CDI_D, CDI_E, HAM-A_1, HAM-A_3, HAM-A_4, HAM-A_5, HAM-A_6, HAM-A_7, HAM-A_11, and HAM-A 14 were significantly reduced in Te+24h compared to T0+2h ($p < 0.001$, $p = 0.014$, $p = 0.009$, $p < 0.001$, $p = 0.005$, $p < 0.001$, $p < 0.001$, $p = 0.005$, $p = 0.005$, $= 0.002$, $p = 0.003$, $p < 0.001$, $p = 0.004$, $p = 0.050$, $p = 0.026$, $p < 0.001$, $p < 0.001$, $p < 0.001$, $p = 0.022$, respectively). All results are summarized in Figure S3.

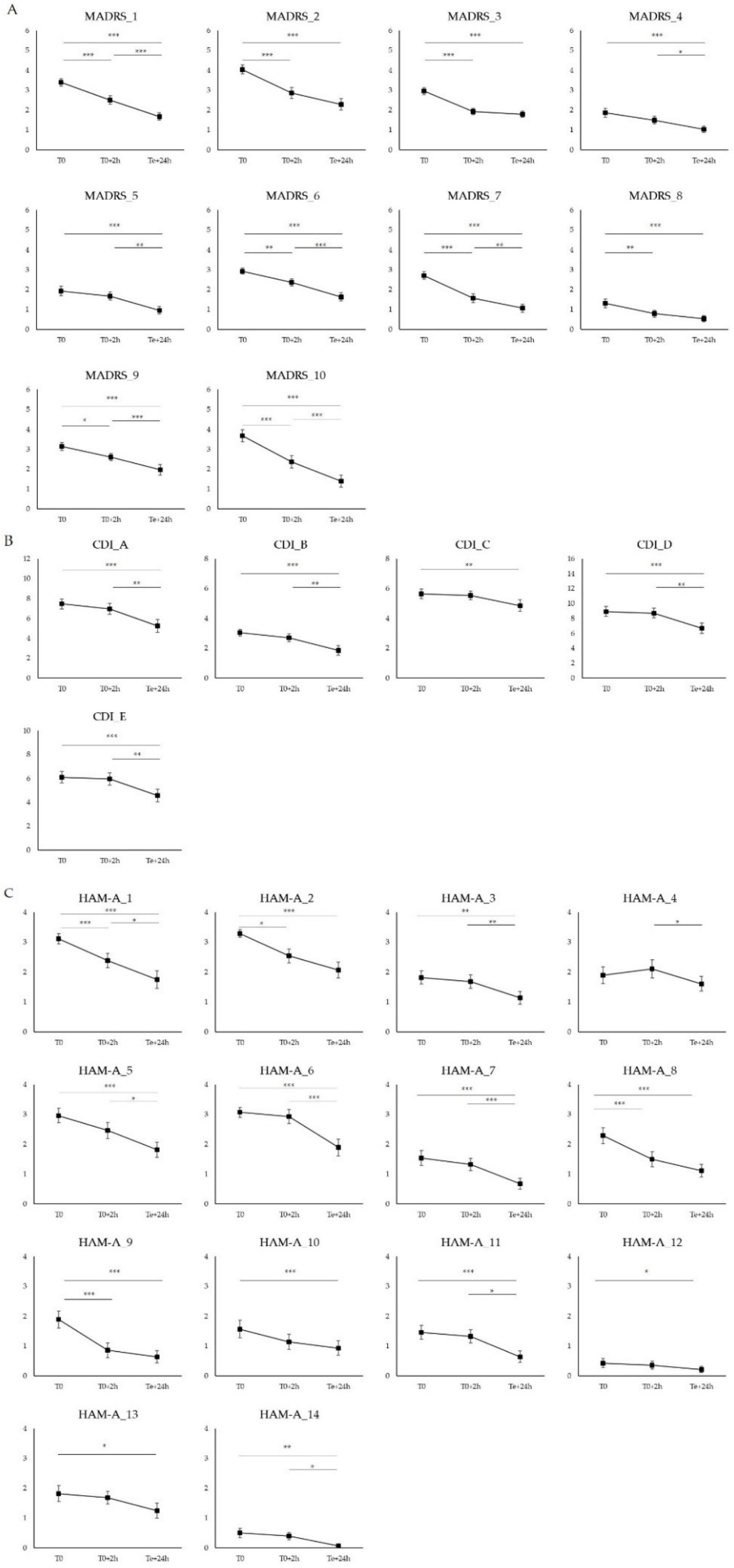
A
MADRS_1
MADRS_2
MADRS_3
MADRS_4
MADRS_5
MADRS_6
MADRS_7
MADRS_8
MADRS_9
MADRS_10
B
CDI_A
CDI_B
CDI_C
CDI_D
CDI_E
C
HAM-A_1
HAM-A_2
HAM-A_3
HAM-A_4
HAM-A_5
HAM-A_6
HAM-A_7
HAM-A_8
HAM-A_9
HAM-A_10
HAM-A_11
HAM-A_12
HAM-A_13
HAM-A_14
T0
T0+2h
Te+24h

Figure S3. Effect of time (T0, T0+2h, Te+24h) within midazolam group. (**A**) MADRS parameters; (**B**) CDI parameters; (**C**) HAM-A parameters. Abbreviations: T0—baseline, i.e. time point before treatment, T0+2h—time point representing two hours after initial infusion, Te+24h—time point representing twenty-four hours after last infusion, MADRS—the Montgomery-Åsberg Depression Rating Scale, CDI—the self-report Children's Depression Inventory, and HAM-A—Hamilton Anxiety Rating Scale. Data are expressed as mean and SEM. * represents statistical significance at level $p < 0.05$; ** represent statistical significance at level $p < 0.01$, *** represent statistical significance at level $p < 0.001$.
